# New synonymies and new combinations of Muscidae from China (Diptera, Muscoidea)

**DOI:** 10.3897/zookeys.290.4811

**Published:** 2013-04-16

**Authors:** Ming-Fu Wang

**Affiliations:** 1 Institute of Entomology, Shenyang Normal University, Shenyang 110034, Liaoning, P. R. China

**Keywords:** New synonymies, new combinations, *Helina*, *Myospila*, *Spilogona*, *Hebecnema*, Muscidae

## Abstract

New synonymies and new combinations are proposed, based mainly on the study of type materials. They are as follows: *Helina sarmentosa* Fang & Fan, 1993 = *Helina dianica* Qian & Feng, 2005, **syn. n.**; *Helina dianxiia* Xue and Li, 2002 = *Helina aureolicolorata* Feng & Xue, 2002, **syn. n.**; *Myospila lenticeps* (Thomson, 1869) = *Helina magnimaculata* Feng, 1995, **syn. n.**; *Spilogona angulisurstyla* (Xue & Xiang, 1998), **comb. n.**; *Spilogona apicicauda *(Xue, Wang & Tong, 2003), **comb. n.**; *Hebecnema arcuatiabdomina* (Feng & Fan, 2001), **comb. n.**

## Introduction

The genus *Helina* Robineau-Desvoidy, 1830 is the second largest genus in the dipteran family Muscidae. It occurs in all zoogeographic regions of the world and comprises over 520 species (Pont 1980, 1986, Carvalho et al. 2005, Wang et al. 2008). According to present data, over 230 species of *Helina* have been found in China (Xue and Chao 1998, Xue et al. 2005, Wang et al. 2004, 2005a, 2005b, 2006a, 2006b, 2008). Early taxonomists, like Stein (1907, 1915), studied *Helina* from Taiwan and Northwest China, which formed the basis for further research on this genus in the Chinese fauna. Further impetus was provided by Emden’s (1965) work on Muscidae for the Fauna of India series. The major references dealing with Chinese *Helina* are Hennig (1957–1958), Ma (1981), Xue and Wang (1982), Fang et al. (1986), Pont (1986), Wu (1989), Xue et al. (2005) and Wang et al. (2004, 2005a, 2005b, 2006a, 2006b, 2008).

*Helina* includes so many species that it is an intimidating task to undertake research on, or even to identify the species correctly. Furthermore, many specialists consider that *Helina* is a ‘‘catch-all’’ repository for species that cannot be assigned elsewhere (Hennig 1965, Wang et al. 2008). The genus needs to be fully revised. In this paper, new synonymies and new combinations are proposed, based mainly on the study of type materials.

## Material and methods

This study has been based on materials from the following museums:

**IESNU** Institute of Entomology, Shenyang Normal University, Shenyang, Liaoning Province, China

**IMEAMMS** Institute of Microbiology and Epidemiology, Academy of Military Medical Sciences, Beijing, China

**IZCAS** Institute of Zoology, Chinese Academy of Sciences, Beijing, China

**SEMCAS** Shanghai Entomological Museum, Chinese Academy of Sciences, Shanghai, China

Morphological terms follow McAlpine (1981), except the “postpedicel” and “prealar setae” follows Stuckenberg (1999) and Fan (1992), respectively. Absolute measurements are used for body length in millimeters (mm). The following abbreviations are used in this article: ***acr***: acrostichal setae; ***dc***: dorsocentral setae; ***ia***: intra-alar setae; ***pra***: prealar setae; ***ad***: anterodorsal setae; ***av***: anteroventral setae; ***p***: posterior setae and ***pv***: posteroventral setae.

## Taxonomy

### Helina sarmentosa

Fang & Fan, 1993

http://species-id.net/wiki/Helina_sarmentosa

Helina sarmentosa Fang & Fan, 1993: 1239; Xue and Chao 1998: 2283; Wang et al. 2004: 297; Xue et al. 2005: 40; Xue and Wang 2006: 178.
Helina dianica Qian & Feng, 2005: 260. **Syn. n.**

#### Redescription.

MALE. Body length 8.5–9.5mm. Eye with dense and long hairs; frons about 1.3–2.0 times as wide as the width of anterior ocellus; frontal setae reaching ocellar triangle; fronto-orbital plate and parafacial with silveryish-grey pollinosity; parafacial bare, about 1.5–2.0 times as wide as postpedicel; antenna black, postpedicel about 2.6–3.0 times as long as broad, about 1.8 times as long as pedicel, arista plumose, the longest hair about equal or slightly longer than postpedicel width; gena and postgena with black hairs, gena with 5 rows of subvibrissal setae in anterior margin, genal height about 2/5 times of eye height; proboscis stout, prementum about 2.0 times as long as height; palpus black, slightly flat in distal half. Thorax ground-color black, scutum with four dark vittae in posterior view, ***acr*** 0+1, 9 rows of irregular fine setae between two ***dc*** rows, ***dc*** 2+4, ***ia*** 0+2, ***pra*** absent; anepisternum with hairs around anepisternal setae, meron with hairs around the lower margin of posterior spiracle, scutellum with black hairs in the lateral margin; basisternum, proepisternum, anepimeron, katepimeron bare; katepisternal setae 2+2, spiracles dark brown. Wing hyaline, brownish, with heavier color in the basal part and around veins; costal spine inconspicuous, radial node bare; veins R_4+5_ and M conspicuously diverging distally, crossveins r-m and dm-cu lightly clouded, crossvein m-m sinuate; calypteres yellowish; halteres brownish-yellow. Legs black; fore tibia with 2–4 ***p***; basal half of mid femur with row of 8–10 ***pv*** (stout towards distally), distal half with 2 or 3 slender setae, mid tibia with 1 or 2 ***ad*** and 6 or 7 developed ***p***, the length of longest seta about 3/5 of width of tibia; hind coxa bare on rear surface, hind femur with 5–7 strong setae on distal half, hind tibia with 7–10 ***av***, 3–5 developed ***ad*** and 6–9 ***p***. Abdomen ground-color black, with dense greyish-blue pollinosity, tergites 2 to 4 with one large dark lateral patch on each side, tergites 4 and 5 with posterior marginal setae, tergite 5 with irregular discal setae, sternite 1 with hairs.

FEMALE. Unknown.

#### Remarks.

Both *Helina sarmentosa* and *Helina dianica* can be easily separated from the other *Helina* species by mid and hind tibiae with very long and stout setae. Having examined the holotypes of both nominal species, we were unable to find differences justifying their separation, which has led us to consider all the examined specimens to be conspecific. Therefore, we synonymized *Helina dianica* under *Helina sarmentosa*.

#### Material studied.

Holotype of *Helina sarmentosa*, ♂: China: Yunnan, Deqin, Mt. Meili, alt. 4,300–4,680m a.s.l., 29.VII.1982, Coll. H.C. Cai(IZCAS); paratype of *Helina sarmentosa*, ♂: China: Yunnan, Deqin, Mt. Meili, alt. 4,300–4,680m a.s.l., 28.VII.1982, Coll. X.Z. Zhang(SEMCAS). Holotype of *Helina dianica*, ♂: China: Yunnan, Deqin, alt. 2,400m a.s.l., 29.VII.1991, Coll. unknown (IMEAMMS).

#### Distribution.

China (Yunnan).

### Helina dianxiia

Xue & Li, 2002

http://species-id.net/wiki/Helina_dianxiia

Helina dianxiia Xue & Li, 2002: 78; Xue et al. 2005: 39; Xue and Wang 2006: 173.
Helina aureolicolorata Feng & Xue, 2002: 261; Xue et al. 2005: 39; Xue and Wang 2006: 172. **Syn. n.**

#### Remarks.

The holotype of *Helina dianxiia* is almost identical to *Helina aureolicolorata*. The only difference between these two species is that the body color of *Helina dianxiia* is darker than *Helina aureolicolorata*. *Helina dianxiia* and *Helina aureolicolorata* are synonymized here because the two holotypes of these two nominal species characters are surprisingly consistent and genitalia are identical. Therefore, we synonymized *Helina aureolicolorata* under *Helina dianxiia*.

#### Material studied.

Holotype of *Helina dianxiia*, ♂: China: Yunnan, Lushui, alt. 2,000m a.s.l., 26.V.1992, Coll. F.H. Li (IESNU). Holotype of *Helina aureolicolorata*, ♂: China: Sichuan, Ya’an, Mt. Zhougong, alt. 1,700m a.s.l., 24.VI.1985, Coll. Y. Feng (IMEAMMS).

#### Distribution.

China (Sichuan, Yunnan).

### Myospila lenticeps

(Thomson, 1869)

http://species-id.net/wiki/Myospila_lenticeps

Helina magnimaculata Feng in Deng and Feng, 1995: 140; Xue et al. 2005: 39; Xue and Wang 2006: 176. **Syn. n.**

#### Remarks.

There are more than fifty species of the genus *Myospila* found in China. The species *magnimaculata*, previously assigned to *Helina*, is in my opinion a *Myospila* due to the following characters: vein M distinctly curved forward at tip; cerci slender and the morphology of genitalia. At the same time, I consider it to be a synonym of *Myospila lenticeps* for these other reasons: scutum with 4 ***dc***; costal spine short but distinct; most part of thorax brown with dense light yellow pollinosity and vein R_4+5_ with fine hairs on the basal part in ventral view. Consequently, *Helina magnimaculata* is a new subjective junior synonym of *Myospila lenticeps*.

#### Distribution.

China (Guangdong, Guizhou, Hunan, Sichuan, Taiwan, Yunnan); Japan; Sri Lanka; India; Indonesia; Malaysia; Philippines; Thailand; Nepal; Kiribati (Christmas Island); Madagascar; Principe.

### Spilogona angulisurstyla

(Xue & Xiang, 1998) comb. n.

http://species-id.net/wiki/Spilogona_angulisurstyla

Helina angulisurstyla Xue & Xiang in Xue and Chao 1998: 1126; Xue et al. 2005: 45; Xue and Wang 2006: 171.

#### Material studied.

Paratype, ♂: China: Xinjiang, Dong Kunlun, Kaerdong, alt. 4,300m a.s.l., 11.VII.1984, Coll. C.Q. Xiang (IESNU); paratype, ♂: China: Xinjiang, Hejing, alt. 3,000–3,500m a.s.l., 31.VII.1958, Coll. C.Q. Li (IZCAS).

#### Remarks.

*Helina angulisurstyla* was described by Xue and Xiang in Xue and Chao (1998) and can be easily distinguished from other *Helina* species by the slightly shortened arista, ***pra*** absent, katepisternal setae 1+1, veins R_4+5_ and M not diverging distally, abdomen with trapezoid patches, sternite 1 bare, sternite 5 broad and short, cerci long and distal sharped, surstyli not expanded distally but sharp in lateral view. However, these diagnostic characters are practically identical with the generic characters of *Spilogona*. Our analysis of the paratypes of *Helina angulisurstyla* revealed a similar external morphology; especially the male genital structures with species in the genus *Spilogona*. Consequently, we suggest the following new combination: *Spilogona angulisurstyla*.

#### Distribution.

China (Xinjiang).

### Spilogona apicicauda

(Xue, Wang & Tong, 2003) comb. n.

http://species-id.net/wiki/Spilogona_apicicauda

Helina apicicauda Xue, Wang & Tong, 2003: 754; Xue and Wang 2006: 171.

#### Material studied.

Holotype, ♂: China: Qinghai, Yushu, Batang, alt. 4,200–4,500m a.s.l., 15.VI.1964, Coll. S.Y. Wang (IZCAS).

#### Remarks.

Diagnostic characteristics (arista short; ***pra*** absent; katepisternal setae 1+2; costa with distinct comb-like rows on the front margin, vein m-m straight; mid tibia with 2 ***ad*** and 2 ***p***; tergites 3 and 4 with one pair dark patches, sternite1 bare; surstyli slender, the lateral margin of cerci curved in distal half, apical of cerci sharp, sternite 5 broad and short) of the holotype indicate that it belongs to *Spilogona*.

#### Distribution.

China (Qinghai).

### Hebecnema arcuatiabdomina

(Feng & Fan, 2001) comb. n.

http://species-id.net/wiki/Hebecnema_arcuatiabdomina

Helina arcuatiabdomina Feng & Fan, 2001: 188; Xue et al. 2005: 47; Xue and Wang 2006: 171.

#### Description.

(Translated from Feng and Fan 2001.) MALE. Body length about 7.0mm. Eye bare, upper part slightly flattened, facets obviously expanded on anterior margin in upper part, frons at its narrowest point about 1/3 of the width of anterior ocellus, lower half of fronto-orbital plate with 3 frontal setae; ocelli mound-like; parafacial about half as wide as postpedicel; antennal scape and pedicel black, brown distally, postpedicel 2.5 times as long as broad, arista short plumose, the longest hairs slightly shorter than half of postpedicel width; face distally slightly raised, upper part with a flat facial ridge; gena and postgena with black hairs, front gena with subvibrissal setae into 2 rows, genal height about 1/10 of the eye height; prementum about as long as high; palpus dark. Thorax with thin greyish blue pollinosity, scutum without distinct longitudinal stripes, ***acr*** 3+5 (prescutellar ***acr*** short), ***dc*** 2+4, ***ia*** 0+2, ***pra*** absent; lateral and lower margins of scutellum bare; basisternum, notopleuron, meron, katepimeron and metanepisternum bare; spiracle dark brown, half-open; proepisternal setae 2, proepimeral setae 2, katepisternal setae 1+2. Tegula blackish-brown, basicosta brownish-yellow, costal spine inconspicuous; node of Rs bare, crossveins without a cloudy appearance, veins R_4+5_ and M slightly diverging in distal part; calypteres brownish, halteres dark redish-brown. Coxae and trochanters brownish-yellow; femora and tibiae brownish-yellow, tarsi black, distal part of tarsi with yellow rings; fore tibia without ***p***; basal third of mid femur with 4 ***pv*** (short and stout distally), mid tibia with 3 ***p***, distally with 1 ***p***; hind coxa bare on posterior surface, hind femur with complete rows of ***av*** and ***p***, 1 ***av*** distally. Abdomen slightly dark yellow, with thin pollinosity, without distinct stripes and round patches, syntergite 1+2, tergites 3 and 4 with narrow posterior marginal stripes, tergites 3 to 5 with median marginal setae; tergite 6 and sternite 1 bare, sternites 2 and 3 square-shaped.

FEMALE. Unknown.

#### Remarks.

The genus *Hebecnema* Schnabl, 1889 resembles the genus *Helina* but differs from it by the following characters: abdomen always without paired spots; fronto-orbital plate narrow, always narrower than antenna width; head in lateral view slightly flat on upper part; surstyli always slender.

According to the original descriptions and illustrations of *Helina arcuatiabdomina* given by Feng and Fan(2001), we can confirm that this species belongs to *Hebecnema*.

#### Distribution.

China (Sichuan).

## Supplementary Material

XML Treatment for Helina sarmentosa

XML Treatment for Helina dianxiia

XML Treatment for Myospila lenticeps

XML Treatment for Spilogona angulisurstyla

XML Treatment for Spilogona apicicauda

XML Treatment for Hebecnema arcuatiabdomina
